# Competing endogenous RNAs (ceRNAs) and drug resistance to cancer therapy

**DOI:** 10.20517/cdr.2024.66

**Published:** 2024-09-25

**Authors:** Kenneth K. W. To, Hang Zhang, William C. Cho

**Affiliations:** ^1^School of Pharmacy, Faculty of Medicine, The Chinese University of Hong Kong, Hong Kong SAR 999077, China.; ^2^Department of Clinical Oncology, Queen Elizabeth Hospital, Hong Kong SAR 999077, China.

**Keywords:** Alternative mRNA polyadenylation, cancer immunotherapy, ceRNA crosstalk, immune evasion, microRNA, mRNA 3’untranslated region, multidrug resistance, non-coding RNA

## Abstract

Competing endogenous RNAs (ceRNAs) are transcripts that possess highly similar microRNA response elements (MREs). microRNAs (miRNAs) are short, endogenous, single-stranded non-coding RNAs (ncRNAs) that can repress gene expression by binding to MREs on the 3’ untranslated regions (UTRs) of the target mRNA transcripts to suppress gene expression by promoting mRNA degradation and/or inhibiting protein translation. mRNA transcripts, circular RNAs (circRNAs), long non-coding RNAs (lncRNAs), and transcribed pseudogenes could share similar MREs, and they can compete for the same pool of miRNAs. These ceRNAs may affect the level of one another by competing for their shared miRNAs. This interplay between different RNAs constitutes a ceRNA network, which regulates many important biological processes. Cancer drug resistance is a major factor leading to treatment failure in patients receiving chemotherapy. It can be acquired through genetic, epigenetic, and various tumor microenvironment mechanisms. The involvement of ceRNA crosstalk and its disruption in chemotherapy resistance is attracting attention in the cancer research community. This review presents an updated summary of the latest research on ceRNA dysregulation causing drug resistance across different cancer types and chemotherapeutic drug classes. Interestingly, accumulating evidence suggests that ceRNAs may be used as prognostic biomarkers to predict clinical response to cancer chemotherapy. Nevertheless, detailed experimental investigations of the putative ceRNA networks generated by computational algorithms are needed to support their translation for therapeutic and prognostic applications.

## INTRODUCTION

**Figure 1:**
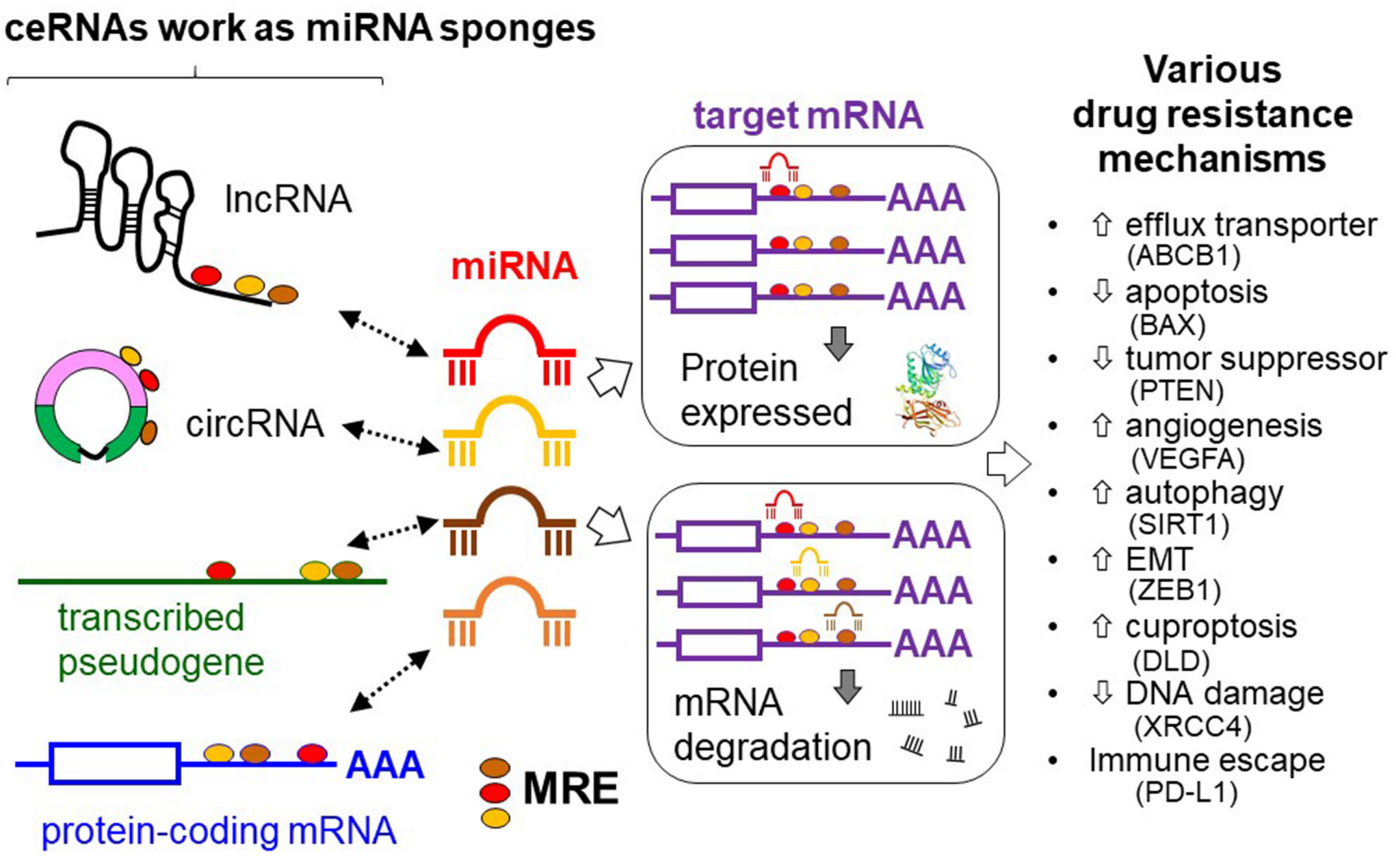
An overview of the dysregulation of ceRNA regulatory network to induce different drug resistance mechanisms (including efflux transporter overexpression, reduced apoptosis, downregulated tumor suppressor, increased angiogenesis, induced autophagy, increased EMT, increased cuproptosis, reduced DNA damage, and immune escape). Representative downstream effectors leading to the different resistance mechanisms are listed in parentheses. RNA molecules could communicate with one another through miRNA and MRE. The expression of one RNA could influence the level and activity of another RNA by sequestering their common miRNA(s). ceRNA: Completing endogenous RNA; EMT: epithelial-mesenchymal transition; miRNA: microRNA; MRE: microRNA response element; circRNA: circular RNA; lncRNA: long non-coding RNA.

microRNAs (miRNAs) are short (~22 nucleotides in length), endogenous, single-stranded non-coding RNAs (ncRNAs) that repress gene expression at the post-transcriptional level in eukaryotic organisms. They bind to miRNA response elements (MREs) on the 3’ untranslated regions (UTRs) of the target mRNA transcripts with imperfect complementarity, subsequently suppressing gene expression by either promoting mRNA degradation or inhibiting protein translation^[1]^. Recent research demonstrated that mRNA transcripts, circular RNAs (circRNAs), long non-coding RNAs (lncRNAs), and transcribed pseudogenes could share highly similar MREs, and they interact with the same set of miRNAs to regulate miRNA activity. These competing endogenous RNAs (ceRNAs) work as decoys for a particular miRNA, thereby abrogating the interaction of the miRNA to its target mRNA^[2-5]^. The expression of ceRNA usually exhibits a negative correlation with the miRNAs that carry the relevant recognition sequences^[6]^. On the other hand, the expression levels of a pair of ceRNAs are usually positively correlated^[7]^. Figure 1 depicts a schematic diagram illustrating the ceRNA networks of mRNA/miRNA/ncRNA to induce cancer drug resistance. Numerous *in silico* algorithms have been established to predict putative pairs of ceRNA in accordance with their shared miRNAs and cellular expression^[8]^.

The ceRNA regulatory network involving different RNA molecules can have significant implications for various biological processes and disease progression. This area of active research is providing insights into the complexity of gene regulation. While most ceRNA interactions reported in the literature reflect only single binding partners (i.e., one miRNA and a pair of ceRNA transcripts), emerging evidence suggests that ceRNA crosstalk occurs in an interconnected manner as a network. Besides direct interactions via shared miRNAs, indirect interactions may also mediate a significant effect on ceRNA modulation. Further investigation of the ceRNA crosstalk should also involve analysis of potential miRNAs and ceRNA networks. Targeting nonessential nodes within the regulatory network will not produce a useful therapeutic response because cancer cells could overcome the damage through alternative survival pathways. Instead, the critical juncture of the ceRNA network may represent potential therapeutic targets for cancer therapy.

Cancer drug resistance is a major unresolved obstacle to successful cancer chemotherapy. It is a multifactorial phenomenon that is mediated by increased drug efflux, reduced susceptibility to apoptosis, accelerated DNA damage repair, aberrant drug biotransformation, mutations in cellular molecular targets, and reduced anticancer immunity^[9]^. Accumulating evidence suggests that dysregulated ceRNA regulation is involved in the drug resistance mechanisms affecting numerous anticancer drugs.

In this review, we present the latest investigation about how ceRNA dysregulation triggers drug resistance to cancer therapy across different cancer types and chemotherapeutic drug classes. Representative preclinical studies and clinical findings are highlighted. Emerging prognostic biomarkers based on ceRNAs to predict clinical response to cancer chemotherapy will also be discussed.

## SPECIFIC CERNA NETWORK MEDIATING DIFFERENT CHEMORESISTANCE MECHANISMS

By sequestering miRNAs and preventing them from interacting with their target mRNAs, ceRNAs are known to indirectly modulate the expression of genes regulating drug efflux, drug biotransformation, apoptosis, DNA repair, and other mechanisms associated with chemoresistance. Liu *et al*. reported the first comprehensive analysis of ceRNAs in drug resistance across different cancer types and anticancer drug classes^[10]^. In their study, using information about the shared miRNAs and correlation of lncRNA and mRNA expressions retrieved from public databases (lncRNA expression data from TANRIC; mRNA expression signature of cancer cell lines from CCLE; regulatory relationship between miRNA-lncRNA and miRNA-gene according to DIANA-LncBase and miRTarBase), a general ceRNA network was first constructed from 183 lncRNAs and 379 mRNAs. Distinct ceRNA modules (encompassing 138 drugs and 19 cancer types) related to cancer drug resistance were identified in 758 drug-cancer conditions, where there are significantly more differentially-expressed lncRNA and mRNA between drug-resistant and -sensitive cancer cell lines^[10]^. Importantly, the functional analysis indicated that resistance-related biological processes (including accelerated cell proliferation, enhanced DNA damage repair, and reduced apoptosis) were enriched in these drug resistance-related ceRNA modules. It is noteworthy that some anticancer drugs (such as the multitargeted kinase inhibitors dasatinib and sunitinib) share the same modules^[10]^. These drugs may exhibit similar drug resistance mechanism(s). Moreover, a Jaccard index (a measure of the similarity of different ceRNA modules) of all clinically approved drugs has been computed to examine the possibility of multidrug resistance (MDR). Anticancer drugs with a high Jaccard index were found to possess similar structural backbones^[10]^. It is logical because drugs with a high chemical similarity will likely display a similar spectrum of therapeutic efficacy and are more likely to be affected by MDR. The information may help clinicians identify a better treatment choice for drug-refractory tumors.

### ceRNA dysregulation induces resistance to traditional cancer chemotherapy

#### Upregulation of MDR efflux transporters

Overexpression of the MDR efflux transporters in cancer cells is a major mechanism causing chemoresistance. To *et al*. published a comprehensive review recently about the regulation of MDR transporters by ncRNAs, which provided the latest update about the significant impact of ncRNAs on chemoresistance in cancer therapy and novel approaches for its circumvention^[11]^. ncRNAs (including miRNA, lncRNA, and circRNA) are important regulators of cancer cell proliferation, apoptosis, and metabolism^[12,13]^. lncRNAs refer to non-coding transcripts longer than 200 nucleotides in length, which are not translated into proteins^[14,15]^. They are involved in the regulation of chromatin structure and transcription, splicing, various forms of RNA processing, editing, localization and stability, and protein translation and localization through interactions with RNA, DNA, and protein^[16-18]^. Numerous lncRNAs are also known to bind with miRNAs, thereby regulating gene expression of important biological processes^[19,20]^. On the other hand, circRNA is a single-stranded ncRNA subfamily in which its 5’and 3’ ends join to form a covalently closed continuous loop^[21]^. In a typical circRNA molecule, the 3’ or 5’ ends normally present in an RNA are joined together to form a closed loop, which makes them stable against RNA exonuclease-mediated degradation^[22]^. Apart from governing transcription, splicing, translation, and post-translational modifications, circRNAs also regulate gene expression by sequestering miRNAs^[23-25]^. To this end, numerous lncRNAs and circRNAs are known to function as molecular decoys to sponge miRNAs and wipe out their interaction with the target mRNAs, subsequently forming ceRNA networks^[26]^. Table 1 summarizes the representative lncRNA/circRNA-miRNA-ABC efflux transporter regulating ceRNA machineries that have been shown to promote MDR to various chemotherapeutic drugs in different cancer types. A few representative examples are highlighted below.

**Table 1:** Representative ceRNA networks promoting overexpression of multidrug resistance ABC efflux transporters in chemoresistant cancer cells

**Efflux transporter**	**Dysregulated ncRNA**	**Anticancer drug**	**Cancer type**	**Ref.**
ABCB1	DANCR (lncRNA; upregulated)	Docetaxel	PCa	[129]
	FENDRR (lncRNA; downregulated)	Doxorubicin	CML	[130]
	FTH1P3 (lncRNA; upregulated)	Paclitaxel	BC	[131]
	GAS5 (lncRNA; downregulated)	Doxorubicin	BC	[34]
	HOTAIR (lncRNA; upregulated)	-	HCC	[132]
	LINC00355 (lncRNA; upregulated)	Cisplatin	Bladder cancer	[133]
	LUCAT1 (lncRNA; upregulated)	Mitoxantrone	OS	[134]
	ROR (lncRNA; upregulated)	Cisplatin	OS	[135]
	SNHG16 (lncRNA; upregulated)	-	CRC	[136]
	UCA1 (lnRNA; upregulated)	Imatinib	Leukemia	[27]
	circ_0004674 (circRNA; upregulated)	Doxorubicin	OS	[137]
ABCC1	CACS15 (lncRNA; upregulated)	Oxaliplatin	CRC	[138]
	circ_0076305 (circRNA; upregulated)	Cisplatin	NSCLC	[139]
	KCNQ10T1 (lncRNA; upregulated)	Oxaliplatin	HCC	[140]
	linc00518 (lncRNA; upregulated)	Doxorubicin	BC	[29]
	linc00707 (lncRNA; upregulated)	Cisplatin	NSCLC	[141]
	NR2F1-AS1 (lncRNA; upregulated)	Oxaliplatin	HCC	[142]
ABCC2	circABCC2 (circRNA; upregulated)	-	HCC	[143]
ABCG2	ANRIL (lncRNA; upregulated)	Cisplatin	Rb	[33]
	circSETD3 (circRNA; upregulated)	Gefitinib	NSCLC	[144]
	HOTAIR (lncRNA; upregulated)	Oxaliplatin	GC	[145]

ceRNA: Competing endogenous RNA; ncRNA: non-coding RNA; lncRNA: long noncoding RNA; PCa: prostate cancer; CML: chronic myeloid leukemia; BC: breast cancer; HCC: hepatocellular carcinoma; OS: osteosarcoma; CRC: Colorectal cancer; circRNA: circular RNA; NSCLC: non-small cell lung cancer; Rb: retinoblastoma; GC: gastric cancer.

In chronic myeloid leukemia (CML) K562 cells, the upregulation of lncRNA UCA1 was shown to increase the MDR transporter ABCB1 expression and induce resistance to the ABCB1 substrate imatinib^[27]^. Mechanistically, lncUCA1 sequestered miR-16 and revoked the miRNA-mediated repression on ABCB1 mRNA. Most recently, this lncUCA1-miR-16-5p-ABCB1 regulatory network was also confirmed to regulate ABCB1 expression in human placental BeWo cells by RNA pull-down assay^[28]^.

In the doxorubicin-resistant breast cancer cell line (MCF-7/ADR), both mRNA and protein expressions of the lncRNA linc00518 and the MDR efflux transporter ABCC1 were found to be significantly upregulated relative to the parental counterpart (MCF-7)^[29]^. Consistent with typical ceRNA pairs, genetic silencing of linc00518 was found to inhibit the ABCC1 expression through upregulation of miR-199a. To this end, the plasma level of circulating miR-199a-5p exhibited a positive correlation with the disease progression of breast cancer patients^[30]^, thus supporting the potential use of linc00518 and miR-199a as novel prognostic biomarkers for drug-refractory breast cancer.

In a glioblastoma tumor xenograft mouse model, the high expression of LINC00479 was found to correlate well with the rapid growth of the tumor^[31]^. Detailed mechanistic investigation revealed that LINC00479 sponged miR-134 to increase c-Myc expression, thereby inducing the MDR transporter ABCC1 and triggering temozolomide resistance^[31]^. This study established a co-expression relationship between MYC and ABCC1, and that overexpression of MYC could increase ABCC1 levels in glioma cells. Moreover, using rescue experiments, the regulation of ABCC1 by LINC00479 was shown to be mediated by miR-134 whereas the regulation of ABCC1 by miR-134 was mediated by MYC, thus verifying the LINC00479/miR-134/MYC/ABCC1 ceRNA network. In gemcitabine-resistant bladder cancer cells, the antisense lncRNA (FOXD1-ASP1) was found to express at a very high level, which sequestered miR-143 to upregulate the MDR transporter ABCC3 expression^[32]^.

It has been reported that the expression of a few chemoresistance-causing lncRNAs was induced by specific transcription/epigenetic factors at the transcription level (i.e., via the lncRNA promoters). In cisplatin-resistant retinoblastoma cells, the lncRNA antisense non-coding RNA in the INK4 locus (ANRIL) was upregulated due to increased binding of the hypoxia-inducible factor (HIF-1α) to the lncRNA promoter^[33]^. The elevated expression of ANRIL subsequently upregulates the MDR efflux transporter ABCG2 by sequestering miR-328^[33]^.

The ceRNA network involving lncRNA and miRNA was also reported to drive the MDR phenotype in cancer patient tumor specimens. The drug resistance mechanism has been studied recently in breast cancer tissues from a cohort of 10 responders and 16 non-responders to a neoadjuvant chemotherapeutic regimen (epirubicin + cyclophosphamide combination followed by docetaxel monotherapy)^[34]^. Compared to the tumor tissues from the responders, those from the non-responders exhibited a remarkably lower expression of the lncRNA growth arrest-specific 5 (GAS5) (reduction by ~60%) but a significantly higher expression of the MDR efflux transporter ABCB1 (by > 2-fold)^[34]^. In a cell culture study, a doxorubicin-resistant breast cancer cell model (MCF-7/ADR) was also found to express significantly higher levels of ABCB1 but lower levels of GAS5 than the drug-sensitive MCF-7 parental cells^[34]^. Mechanistic investigation revealed that lncRNA GAS5 upregulated Dickkopf-2 (DKK2), thereby sequestering miR-221-3p and inhibiting the Wnt/β-catenin pathway^[34]^. To this end, β-catenin and transcription factor 4 (TCF4) were known to promote ABCB1 mRNA transcription by interacting with the *ABCB1* promoter^[35]^. Thus, reduced expression of GAS5 in MDR breast cancer cells could upregulate Wnt/β-catenin signaling and induce the MDR transporter ABCB1 expression.

Apart from the regulation of MDR transporters by ceRNAs, other key chemoresistance mediators are also induced by the dysregulation of ceRNA networks, as described below. Table 2 summarizes various other cancer drug resistance mechanisms triggered by ceRNA dysregulation, which affect different classes of conventional chemotherapeutic anticancer drugs. Key clinical findings are summarized in Table 3.

**Table 2:** Representative ceRNA networks promoting the various chemoresistance mediators in preclinical studies

**Chemoresistance mediator (effector gene)**	**Anticancer drug(s)**	**Dysregulated ncRNA**	**Cancer type**	**Ref.**
Apoptosis (BAX)	Cisplatin	lncRNA XIST (upregulated)	NSCLC	[39]
Apoptosis (Aurora B kinase)	Cisplatin	lncRNA XIST (upregulated)	GC	[146]
Autophagy (SIRT1)	5-FU	lncRNA H19 (upregulated)	CRC	[61]
Autophagy (STX17, RAB33B, UVRAG)	Paclitaxel	Protein-coding mRNA SLC7A11 (downregulated)	OC	[147]
Cell cycle regulation (CCND1)	Doxorubicin	lncRNA PVT1 (upregulated)	OS	[148]
Cell proliferation (KPNA4)	Paclitaxel	circRNA circ_ZFR (upregulated)	NSCLC	[40]
Cuproptosis (DLD)	Tamoxifen	lncRNA C6orf99 (upregulated)	TNBC	[68]
DNA damage response/G2 cell cycle checkpoint (WEE1)	Temozolomide	lncRNA FOXD3-AS1 (upregulated)	GBM	[149]
EMT	Oxaliplatin	lncRNA H19 (upregulated under hypoxic condition)	CRC	[62]
EMT (ZEB1)	Doxorubicin	lncRNA HULC (upregulated)	HCC	[63]
Glutamine metabolism (GLS)	Cisplatin	lncRNA NEAT1 (upregulated)	MB	[150]
Glycolysis (GOT1)	Cisplatin	circRNA circGOT1 (upregulated)	ESCC	[151]
Mitophagy (p62)	Lenvatinib	lncRNA LINC01607 (upregulated)	HCC	[152]
Senescence (SALL1, METAP1, DCAF11)	Sunitinib	lncRNA LINC00461 (upregulated)	RCC	[153]
Tumor suppression (PTEN)	Cisplatin, paclitaxel, and docetaxel	lncRNA HOTAIR (upregulated)	CC	[38]
Transcription factor critical for the Hippo pathway (TEAD1)	Gemcitabine	lncRNA MKLN1-AS (upregulated)	PC	[154]
Tyrosine kinase (BCR-ABL)	Imatinib	circRNA circCRKL (upregulated)	CML	[155]

ceRNA: Competing endogenous RNA; ncRNA: non-coding RNA; lncRNA: long non-coding RNA; NSCLC: non-small cell lung cancer; GC: gastric cancer; 5-FU: 5-fluorouracil; CRC: colorectal cancer; OC: ovarian cancer; OS: osteosarcoma; TNBC: triple negative breast cancer; GBM: glioblastoma; EMT: epithelial mesenchymal transition; HCC: hepatocellular carcinoma; MB: medulloblastoma; ESCC: esophageal squamous cell carcinoma; RCC: renal cell carcinoma; CC: cervical cancer; PC: pancreatic cancer; CML: chronic myeloid leukemia.

**Table 3:** Representative clinical data showing the significance of ceRNA dysregulation in mediating cancer drug resistance

**Cancer type**	**Dysregulated ncRNA**	**miRNA involved**	**mRNA target of miRNA**	**Ref.**
BC	lncRNA GAS5 (lower expression in chemotherapy (epirubicin + cyclophosphamide combination)-refractory patients)	miR-221-3p	ABCB1 (efflux transporter-mediated resistance)	[34]
BC	lncRNA C6orf99 (highly expressed in ER+ BC patients resistant to endocrine therapy)	miR-370-3p, miR-432-5p	DLD (cuproptosis)	[67]
BC	lncRNA LINC00589 (can be used as an independent prognostic factor for discriminating tratuzumab responders)	miR-100 and miR-452	DLG5 and PRDM16 (tumor suppressor)	[80]
CML	lncRNA HULC (elevated in CML patients with advanced clinical stages)	miR-200a	c-myc and Bcl-2 (apoptosis)	[85]
CRC	circNCOA3 (overexpressed in patients resistant to anti-PD-1 mAb)	miR-203-3p	CXCL1 (immune checkpoint)	[107]
CRC	Exosomal ciRS-122 (elevated in serum samples of oxaliplatin refractory CRC patients)	miR-122	PKM2 (glycolysis)	[70]
CRC	H19 (increased in recurrent patients)	miR-194-5p	SIRT1 (autophagy)	[61]
ESCC	Exosomal lncRNA PART1 (elevated serum level in patients demonstrating poor response to geftinib)	miR-129	Bcl-2 (apoptosis)	[87]
GBM	Exosomal lncRNA SBF2-AS1 (elevated in serum sample of temozolomide resistant patients)	miR-151a	XRCC4 (DNA double strand break repair)	[75]
HCC	HULC (highly upregulated in patients with more advanced TNM staging)	miR-200a-3p	ZEB1 (EMT)	[63]
NSCLC	circCPA4 (low level in tumor) associated with better prognosis in patients on PD-L1 immunotherapy	let-7	PD-L1 (immune checkpoint)	[106]

ceRNA: Competing endogenous RNA; ncRNA: non-coding RNA; miRNA: microRNA; BC: breast cancer; lncRNA: long non-coding RNA; ER+: estrogen receptor-positive; CML: Chronic myeloid leukemia; CRC: colorectal cancer; DLD: dihydrolipoamide dehydrogenase; PKM2: pyruvate kinase M2; ESCC: esophageal squamous cell carcinoma; GBM: glioblastoma; HCC: hepatocellular carcinoma; EMT: epithelial mesenchymal transition; NSCLC: non-small cell lung cancer.

#### Inhibition of apoptosis

The PI3K/Akt/mTOR signaling pathway plays a central role in regulating cancer cell survival, proliferation, and apoptosis^[36]^. PTEN is an important tumor suppressor that inhibits PI3k/Akt/mTOR to promote apoptosis^[37]^. In cervical cancer, Zhang *et al*. reported that the lncRNA HOTAIR could sponge miR-29b, which indirectly inhibited PTEN by upregulating SP1 expression^[38]^. Interestingly, miR-29b did not affect promoter methylation of PTEN, but it regulated PTEN by targeting the SP1 transcription factor. HOTAIR was shown to induce resistance of cervical cancer cell lines HeLa and Siha to cisplatin, docetaxel, and paclitaxel, which could be reversed by miR-29b upregulation. In non-small cell lung cancer (NSCLC), the lncRNA XIST was reported to sponge miR-520 and induce cisplatin resistance by regulating the apoptotic gene BAX via the p53 signaling pathway^[39]^. Recently, a circRNA circ_ZFR was reported to be highly upregulated in paclitaxel-resistant NSCLC tumor specimens and cell lines^[40]^. To this end, circ_ZFR knockdown was shown to overcome paclitaxel resistance by reducing KPNA4 expression and inducing apoptosis via sponging miR-195-5p.

#### Inhibition of tumor suppressor genes

The loss of function of tumor suppressor genes is well known to cause cancer drug resistance^[41]^. Phosphatase and tensin homolog on chromosome 10 (PTEN) is an extensively studied tumor suppressor^[42]^. PTEN deficiency and its dysfunction leads to aggressive tumor phenotype and reduced response to anticancer therapy. Tay *et al*. were one of the first research teams to identify endogenous protein-coding transcripts (SERINC1, VAPA, CCR4-NOT, and CNOT6L) as PTEN ceRNAs that regulate PTEN expression in a miRNA-dependent manner^[43]^. As PTEN and its ceRNAs share the same miRNAs, deletion of individual ceRNA by siRNAs allowed more miRNAs to interact with PTEN 3’ untranslated region (3’UTR), thereby reducing PTEN mRNA and protein levels. Moreover, the mutual reciprocal regulation of PTEN and its ceRNAs was demonstrated by the fact that PTEN downregulation modulates its ceRNA expression and PTEN ceRNAs are coexpressed with PTEN in patient specimens^[43]^. Pseudogenes are DNA sequences that share high sequence similarity to a known gene, but they are not coded into proteins^[44]^. Due to the high homolog between pseudogenes and their protein-coding partners, pseudogenes may sequester the shared miRNAs and prevent them from binding to their authentic protein-coding transcripts. A representative example is PTENP1, the pseudogene of PTEN, which competes with PTEN for the same set of miRNAs through numerous conserved MREs^[45]^. The direct association between PTENP1 and PTEN expression in tumor specimens from colon cancer patients suggests that PTENP1 transcript may act as a tumor suppressor by regulating PTEN expression^[45,46]^. Intriguingly, PTENP1 is known to produce both sense and antisense transcripts to modulate PTEN expression. The PTENP1 sense transcript shares high sequence similarity with PTEN, thus competing with PTEN for binding specific miRNAs to affect PTEN expression. Meanwhile, two functionally independent isoforms (PTENP1-AS-α and PTENP1-AS-β) are transcribed from the antisense strand. PTEN1-AS-α has been shown to bind to the 5’UTR (promoter) of PTEN, which recruits the epigenetic modifiers (including EZH2 and DNMT3A) to suppress PTEN transcription. On the other hand, PTEN1-AS-β was known to bind to the PTEN1 sense transcript, thus forming a complex that is exported to the cytoplasm. As PTEN1-AS-β lacks a poly-A tail, it stabilizes the PTENP1 sense transcript, which works as a miRNA sponge to post-transcriptionally regulate PTEN.

Recent studies reported an intriguing enrichment of 3’UTR shortening among transcripts that could serve as ceRNAs for tumor suppressor genes^[47]^. The 3’UTR of mRNA contains recognition sequences for various regulatory elements, including miRNA binding sites. Interestingly, some mRNA transcripts display differential 3’UTR length. This could be mediated by alternative polyadenylation (APA), alternative splicing, or other mechanisms^[48]^. The 3’UTR length could influence the availability of miRNA binding sites and, therefore, disrupt the ceRNA regulatory network^[47,49]^.

Theoretically, long 3’UTRs tend to harbor more miRNA binding sites and provide more opportunities for miRNA-mediated regulation. It follows that ceRNAs with longer 3’UTRs may have a higher chance to compete for shared miRNAs, thereby sequestering them and affecting the expression of the target genes. This could lead to complex ceRNA networks and regulatory interactions. On the other hand, shorter 3’UTRs usually possess fewer miRNA binding sites, thus limiting ceRNA regulatory potential. This could lead to reduced competition for miRNAs and thus minimize the effect on target gene expression. The relevance of differential mRNA 3’UTR length in ceRNA regulation lies in its effect on modulating the availability of miRNA binding sites and shaping the ceRNA network^[48]^.

Recent transcriptome-wide studies have revealed that more than 70% of human genes exhibit APA^[50,51]^. As a result of APA, numerous genes have been reported to generate multiple mRNA isoforms bearing differential 3’UTR length, where key RNA regulatory elements are found^[52]^. Mounting evidence suggests that APA can give rise to differential 3’UTR usage according to tissue type, cell state, and environmental condition, thereby coordinating post-transcriptional regulation of numerous genes driving the carcinogenesis process^[53,54]^. To this end, mRNAs harvested from highly proliferative or tumorigenic cells have shorter 3’UTRs, which enables their escape from miRNA-mediated repression of mRNA stability and protein synthesis^[50]^. A widespread recurrent tumor-specific APA regulation in multiple cancer types has recently been identified^[55]^. The prevailing hypothesis is that 3’UTR shortening could activate proto-oncogenes to promote carcinogenesis through the escape of miRNA-mediated repression. Interestingly, according to a recent study correlating the sensitivity of anticancer drugs with APA events characterized by RNA-seq data from The Cancer Genome Atlas (TCGA) data sets^[56]^, the top drug class correlated with global 3’UTR shortening are DNA topoisomerase I inhibitors that are commonly used in treating colorectal cancer.

**Figure 2:**
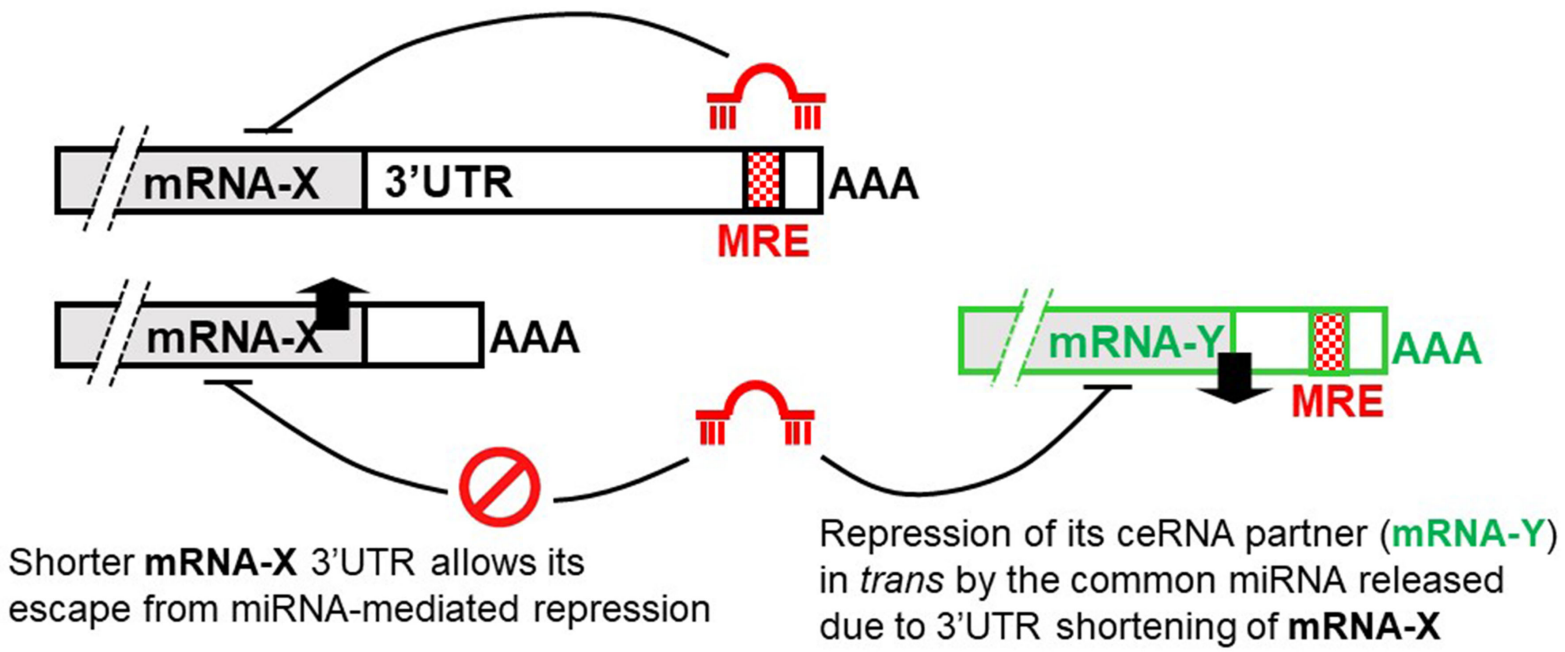
Schematic diagram illustrating how mRNA 3’UTR shortening represses its ceRNA partner in *trans* by releasing the common miRNA(s). 3’UTR shortening of mRNA-X allows its escape from miRNA-mediated repression, therefore increasing mRNA-X expression. However, the release of the common miRNA(s) from binding to the long 3’UTR of mRNA-X will result in the repression of the ceRNA partner mRNA-Y. 3’UTR: 3’ untranslated region; ceRNA: completing endogenous RNA; miRNA: microRNA.

When mRNA transcripts with shortened 3’UTRs lose miRNA recognition sites and no longer sponge the miRNAs, the released miRNAs become available to repress their ceRNA partners [Figure 2]. In various cancer types, the disruption of ceRNA crosstalk by mRNA 3’UTR shortening has been reported for tumor suppressor genes^[47,57,58]^. Intriguingly, genetic silencing of the master 3’UTR-shortening regulator NUDT21 was shown to repress a few tumor suppressor genes (including PHF6 and LARP1) in a ceRNA-dependent manner and promote cancer proliferation^[47]^. Mechanistically, two miRNAs (miR-3187-3p and miR-549) targeting PHF6 were shown to participate in the ceRNA crosstalk. As depletion of DICER1 could abolish PHF6 and NUDT21 crosstalk, the regulation is miRNA-dependent. Moreover, PHF6 3’UTR-mediated luciferase activity was shown to be rescued by the miR-3187-3p antagomir or NUDT21 siRNA.

#### Promotion of tumor angiogenesis

In lung cancer, the lncRNA H19 was known to promote tumor angiogenesis by regulating the anti-angiogenic miRNAs^[59]^. Interestingly, expression of the lncRNA H19 is often induced by cigarette smoke. This smoking-associated H19 dysregulation was shown to provoke angiogenesis by altering the expression of numerous miRNAs (including miR-29, miR-30a, miR-107, miR-140, miR-148b, miR-199a and miR-200) and lead to chemoresistance by inhibiting BiP, DLL4, HIF1α, PDGFB, PDGFRA, and VEGFA^[59]^. Additionally, H19 was also shown to induce tumor-specific pyruvate kinase M2 (PKM2), which is pivotal for the Warburg effect and tumor angiogenesis^[59]^.

#### Induction of autophagy

Autophagy is a cellular degradation and recycling process that plays a dual role in cancer by functioning as a cell survival or death mechanism^[60]^. The dysregulation of ceRNAs has been linked with drug resistance by inducing autophagy. In CRC, the expression of H19 was found to be remarkably increased in recurrent CRC patient tumor samples^[61]^. H19 was demonstrated to induce autophagy via SIRT1 to cause 5-fluorouracil (5-FU) resistance. The overexpression of H19 was shown to promote the conversion of LC3-I to LC3-II, thus leading to a remarkable increase in LC3 aggregation and autophagosome formation but downregulation of the autophagy receptor protein p62 in 5-FU-resistant CRC cells. SIRT1 is a direct target of miR-194-5p. Mechanistically, H19 was shown to sponge miR-194-5p, as demonstrated by gene reporter and immunoprecipitation assays, to induce SIRT1 expression in 5-FU-resistant CRC cells^[61]^.

#### Induction of epithelial-mesenchymal transition

In CRC, the expression of the lncRNA H19 was induced under hypoxic conditions and in oxaliplatin-resistant cells^[62]^. H19 was shown to work as a ceRNA of miR-675-3p to regulate epithelial-mesenchymal transition (EMT) and lead to chemoresistance. Importantly, H19 downregulation could overcome hypoxia-induced chemoresistance by sequestering miR-675-3p to regulate EMT^[62]^.

In HCC, the lncRNA HULC is highly upregulated, which is associated with advanced TNM staging, metastases, recurrence, and poor drug response^[63]^. HULC was shown to sequester miR-200a-3p, which targets and inhibits the transcription factor ZEB1. ZEB1 is critical for EMT and its overexpression is known to drive tumor progression, thus upregulating ZEB1 to mediate EMT and promote metastasis and chemoresistance^[63]^.

In cervical cancer, HOTAIR was found to promote EMT and induce chemoresistance via a miR-29b/PTEN/PI3K ceRNA network^[38]^. HOTAIR was shown to bind with miR-29b. Consistent with the ceRNA mechanism, the expression of HOTAIR and miR-29b is negatively correlated with each other in cervical cancer. While HOTAIR induced cell migration and chemoresistance to cisplatin, docetaxel, and paclitaxel, miR-29b was shown to inhibit EMT. Specifically, miR-29b mimics were shown to downregulate PI3K and enhance cancer drug response.

Recently, a tamoxifen resistance-related ceRNA network has been constructed and validated for breast cancer exhibiting enhanced migration and invasion (EMT phenotype)^[64]^. Differentially expressed mRNAs (DEmRNAs) were screened from drug-resistant breast cancer cells by GEO2R. The 20 top-ranked DEmRNAs were associated with 113 upstream miRNAs and 501 lncRNAs. Among these mRNA/miRNA/lncRNA, 7 mRNAs, 22 lncRNAs, and 11 miRNAs were selected to construct a ceRNA network contributing to tamoxifen resistance in breast cancer. Ultimately, after incorporation of data from GEPIA differential gene expression and Kaplan-Meier survival analyses, 4 mRNAs, 4 lncRNAs, and 3 miRNAs were found to be significantly associated with poor drug response and prognosis^[64]^. The differential gene expression was confirmed by quantitative real-time PCR analysis, thus verifying the novel therapeutic targets for tamoxifen resistance in the constructed ceRNA network.

#### Induction of cuproptosis

Cuproptosis is a new copper metabolism-dependent cell death mechanism^[65,66]^. In triple-negative breast cancer, oral administration of the bioavailable copper chelator tetrathiomolybdate has been shown to deplete mitochondrial copper content and reduce cellular energy production, which is significantly correlated with favorable patient survival^[67]^. A ceRNA network has been constructed using differentially expressed genes related to cuproptosis in estrogen receptor-positive (ER+) breast cancer^[68]^. DLD was found to be a critical cuproptosis-related gene in ER+ breast cancer resistance to endocrine therapy by evaluating the intersection of the protein-protein interaction analysis, differentially expressed genes between the sensitive breast cancer cell lines, and the prognostic cuproptosis-related genes (CRGs)^[68]^. The lncRNA C6orf99 was predicted to be a ceRNA that regulates DLD by sponging hsa-miR-370-3p and hsa-miR-432-5p.

#### Induction of glycolysis

Cancer cells are known to undergo metabolic reprogramming, including increased glycolysis, to promote cell proliferation, metastasis, and chemoresistance^[69]^. In CRC, Wang *et al*. reported that the circRNA ciRS-122 was highly upregulated in oxaliplatin-resistant cells^[70]^. CiRS-122 was shown to sponge miR-122, thus upregulating human pyruvate kinase M2 (PKM2) to promote glycolysis and drug resistance^[70]^. Exosomes are small membrane-bound extracellular vesicles secreted from various cell types. They carry specific nucleic acids, metabolites, and cellular proteins to facilitate intracellular communication^[71]^. Numerous ncRNA are secreted in tumor-derived exosomes and transferred to neighboring cancer cells to mediate the chemoresistance phenotype via a ceRNA mechanism^[40,72,73]^. Importantly, in oxaliplatin-refractory CRC patients, a high expression level of exosomal ciRS-122 in serum samples was significantly associated with unfavorable clinical response to chemotherapy. Moreover, the delivery of siRNA against ciRS-122 via exosomes was shown to reverse oxaliplatin resistance in cell culture and tumor-bearing mouse models by inhibiting the ciRS-122/miR-122/PKM2 ceRNA network, thus inhibiting glycolysis and drug resistance^[70]^. Collectively, drug-resistant cancer cells may exploit ciRS-122 to transfer the chemoresistance phenotype via exosomes to neighboring sensitive cells. Moreover, targeting ciRS-122 may be a novel strategy to overcome oxaliplatin resistance.

#### Alteration of DNA damage response

In ovarian cancer, the lncRNA urothelial carcinoma associated 1 (UCA1) was reported to mediate cisplatin resistance via transfer in tumor cells-derived exosomes^[74]^. Mechanistic investigation indicated that UCA1 worked as a ceRNA of miR-143 to increase the expression of its target FOS-like 2 (FOSL2) mRNA, thereby promoting DNA repair and the drug resistance phenotype. In glioblastoma patients, high serum exosomal lncRNA SBF2-AS1 was shown to contribute to temozolomide resistance^[75]^. Mechanistically, SBF2-AS1 was shown to sequester miR-151a to increase the expression of X-ray repair cross-complementing 4 (XRCC4), thus promoting repair of DNA double-strand break and leading to temozolomide resistance^[75]^.

### ceRNA dysregulation induces resistance to targeted cancer therapy

Targeted cancer therapy refers to the approach that specifically inhibits key molecular signaling pathway(s) to elicit the anticancer effect. Numerous small molecule tyrosine kinase inhibitors have been developed as targeted cancer drugs, which have revolutionized the practice of oncology^[76]^. However, most cancer patients receiving targeted cancer therapy inevitably relapse and develop resistance to the treatment. Various mechanisms are known to contribute to targeted drug resistance, including direct target reactivation, aberrant activation of downstream oncogenes, engagement of parallel oncogenic pathways, and other adaptive cancer survival mechanisms^[77]^. Emerging evidence suggests that ceRNA crosstalk plays a critical role in drug resistance to many targeted anticancer drugs.

A representative example of ceRNA-mediated drug resistance to targeted chemotherapy is the interaction between the lncRNA H19 and the tumor suppressive miR-200a in breast cancer. The role of the lncRNA H19 in various cancer hallmarks has been recently reviewed by Hashemi *et al*.^[78]^. The biological function of H19 in most cancer types is oncogenic, and therefore, high H19 expression is generally correlated with enhanced tumor growth, cell cycle progression, EMT induction, and elevated metastasis. H19 is also well known to trigger chemo- and radio-resistance in cancer cells. Numerous downstream target genes and molecular pathways for lncRNA H19 have been identified and validated, which include miRNAs, RUNX1, STAT3, β-catenin, and FOXM1. To this end, H19-miR-200a constitutes a ceRNA network to mediate resistance to the targeted chemotherapy drug trastuzumab, commonly used for treating HER2-positive breast cancer^[79]^. H19 acts as a ceRNA to sequester miR-200a, thus preventing it from binding to its target mRNAs ZEB1 and ZEB2. ZEB1 and ZEB2 are important transcription factors involved in the EMT process, which is associated with increased tumor invasiveness and chemoresistance. The increased expression of ZEB1 and ZEB2 promotes EMT and contributes to drug resistance. Inhibition of H19 or ectopic expression of miR-200a could sensitize trastuzumab-resistant breast cancer cells^[79]^.

Bai *et al*. recently reported another LINC00589-dominated ceRNA network regulating resistance to HER2-targeted therapy, cancer stemness properties, and multidrug resistance in breast cancer^[80]^. The lncRNA LINC00589 was reported as an independent prognostic factor for identifying trastuzumab (HER2 inhibitor) responders. Intriguingly, LINC00589 was found to simultaneously sponge miR-100 and miR-452 to relieve their repression of two tumor suppressors (DLG5 and PRDM16), thereby inhibiting cancer proliferation and counteracting drug resistance^[80]^. The two novel ceRNA networks (LINC00589/miR-100/DLG5 and LINC00589/miR-452/PRDM16) may be exploited as useful prognostic markers and novel therapeutic targets for drug-refractory HER2-positive breast cancer.

Sorafenib (a multikinase inhibitor of VEGFR and PDGFR) is the targeted drug of choice for HCC. The let-7 family of miRNAs has been shown to inhibit the expression of the antiapoptotic protein Bcl-xL and potentiate sorafenib-induced apoptosis in HCC^[81]^. It has been proposed that the antitumor efficacy of sorafenib could be compromised by high H19 levels by sponging miR-let-7^[82]^. Another lncRNA NEAT1 was also reported to promote resistance of HCC to sorafenib by sponging miR-355, thus relieving sorafenib-induced inhibition of the c-Met/Akt pathway^[83]^. On the other hand, NEAT1 could also work as a ceRNA of miR-204 to increase the expression of the Autophagy Related 3 (ATG3), subsequently inducing autophagy and leading to sorafenib resistance^[84]^.

In CML, the lncRNA HULC was shown to promote imatinib resistance by a miR-200a/c-myc/Bcl-2 ceRNA regulatory network^[85]^. HULC was shown to be remarkably overexpressed in both leukemia cell lines and hematopoietic cells from CML patients. The elevated HULC level was remarkably associated with more advanced clinical stages of CML. Importantly, HULC silencing was shown to inhibit the activation of PI3K and AKT, and potentiate imatinib-induced apoptosis, which was associated with the downregulation of c-Myc and Bcl-2. Mechanistically, HULC was shown to modulate c-Myc and Bcl-2 by sponging miR-200a^[85]^.

NcRNAs are also transferred in tumor-derived exosomes to mediate targeted drug resistance via ceRNA-related mechanisms. In NSCLC cells, tumor-derived exosomal circRNA_102481 has been reported to mediate EGFR-TKI resistance by sponging miR-30a-5p to modulate ROR1^[86]^. Interestingly, high expression of circRNA_102481 in exosomes isolated from the peripheral blood of NSCLC patients was positively correlated with more advanced TNM stage, poor tumor differentiation status, prominent brain metastasis, and dismal progression-free survival and overall survival. Genetic silencing of circRNA_102481 or administration of exosomes encapsulated with si-circRNA_102481 could inhibit EGFR-TKI resistance and promote apoptosis. Therefore, exosomal circRNA_102481 may represent a useful diagnostic biomarker and novel therapeutic target for EGFR-TKIs resistance in NSCLC. In esophageal squamous cell carcinoma (ESCC), the lncRNA PART1 was enriched in exosomes and it acted as a ceRNA of miR-129 to upregulate Bcl-2 and promote gefitinib resistance^[87]^. Importantly, a high serum level of exosomal PART1 was found to be significantly associated with poor response to gefitinib therapy in ESCC patients. In advanced renal cell carcinoma, exosome-transmitted lncRNA Activated in RCC with Sunitinib Resistance (lncARSR) was shown to mediate sunitinib resistance by sponging miR-34/miR-449 to increase AXL and c-Met expression^[88]^. The lncRNA urothelial carcinoma associated 1 (UCA1) was enriched in ovarian cancer-derived exosomes, and it was reported to mediate gefitinib resistance by sequestering miR-143 to increase FOSL2 expression^[89]^.

### ceRNA dysregulation induces resistance to cancer immunotherapy

There is accumulating evidence to suggest the potential impact of ceRNA regulation on cancer immunotherapy response. The regulation of ceRNAs could affect cancer immunotherapy by regulating the immune checkpoint molecules. Jiang *et al*. recently published a comprehensive review of the regulatory role of lncRNAs and circRNAs in the PD-1/PD-L1 pathway and the relevance to the efficacy of immune checkpoint inhibitors^[90]^. Programmed cell death protein (PD-1) and cytotoxic T-lymphocyte-associated protein 4 (CTLA-4) are the two most extensively studied immune checkpoint molecules that are exploited by cancer cells to evade immune surveillance^[91]^. Immune checkpoint blockade with anti-CTLA-4 (ipilimumab and tremelimumab), anti-PD-1 (nivolumab and pembrolizumab), or anti-PD-ligand (PD-L1) (durvalumab, atezolizumab, and avelumab) monoclonal antibodies is revolutionizing cancer therapy, which induces durable tumor responses and overall survival benefit in a wide variety of cancer types^[92]^. However, some patients do not respond to or develop resistance to immune checkpoint inhibitors, such as anti-PD-1 antibodies. Understanding the mechanisms underlying drug resistance in immunotherapy is crucial for improving patient outcomes.

PD-1 is an inhibitory receptor present on activated immune cells, including T cells, B lymphocytes, natural killer cells, macrophages, dendritic cells, and monocytes, which normally function to blunt both adaptive and innate immune responses. Upon binding of PD-1 to its major ligand PD-L1, which is expressed on tumor cell surface, T cell antitumor immunity will be suppressed. The anti-PD-1/PD-L1 antibodies were designed as immune checkpoint inhibitors for cancer therapy. They work by binding to inhibitory PD-1 receptors on tumor-reactive T cells and PD-L1 on tumor cells, respectively, thereby disrupting the PD-1/PD-L1 interaction and reactivating the antitumor T cell-mediated cell cytotoxicity. Cancer patients with heavy tumor mutational burden, abundant pre-treatment tumor-infiltrating T cells, and elevated pre-treatment PD-L1 levels on tumor cells are expected to respond more favorably to anti-PD-1/PD-L1 immunotherapy^[93]^.

**Figure 3:**
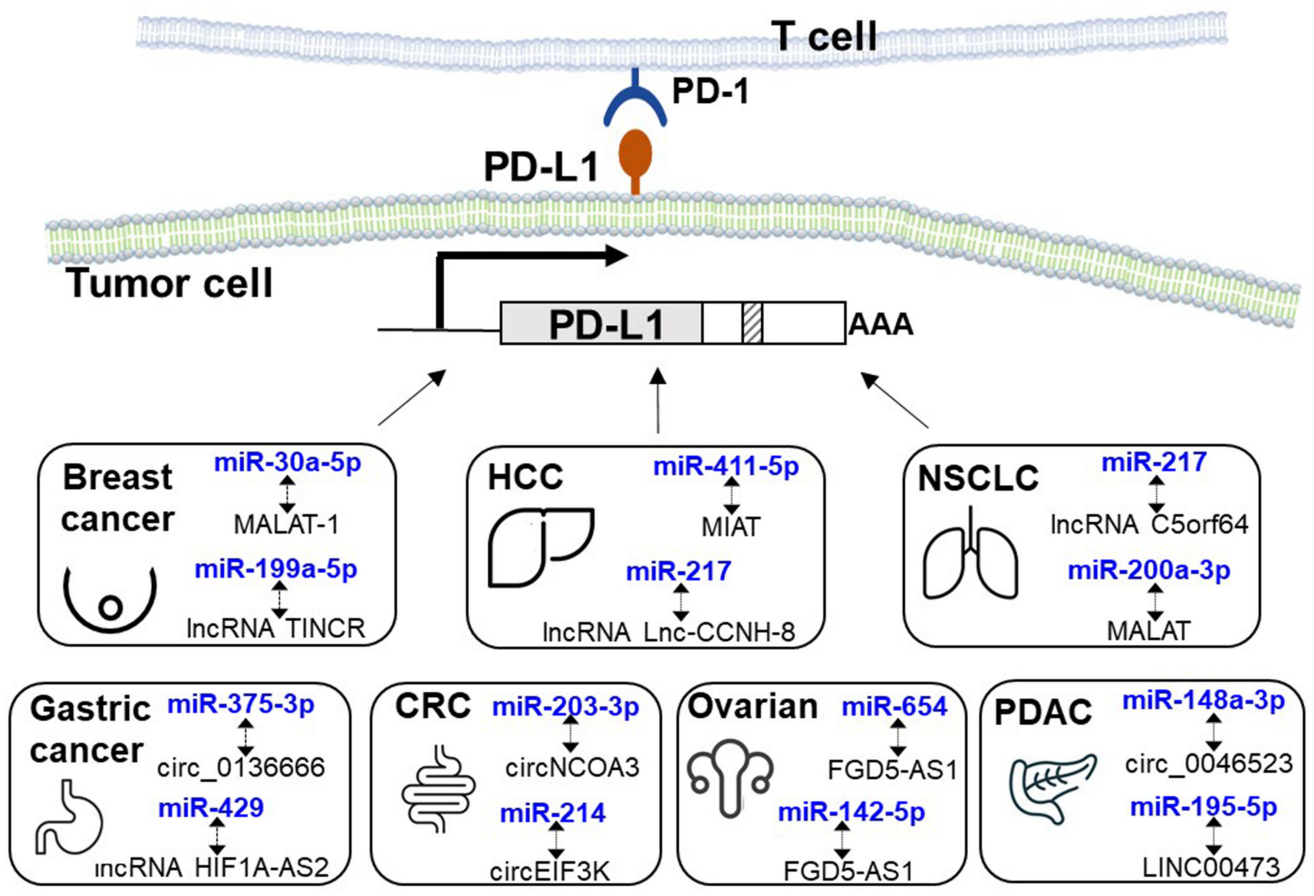
Resistance to cancer immunotherapy due to upregulation of PD-L1 mediated by ceRNA dysregulation in different cancer types. Representative ceRNA networks (miRNA - ncRNA interaction) are shown. PD-L1: Programmed cell death ligand 1; ceRNA: completing endogenous RNA; miRNA: microRNA; ncRNA: non-coding RNA.

**Figure 4:**
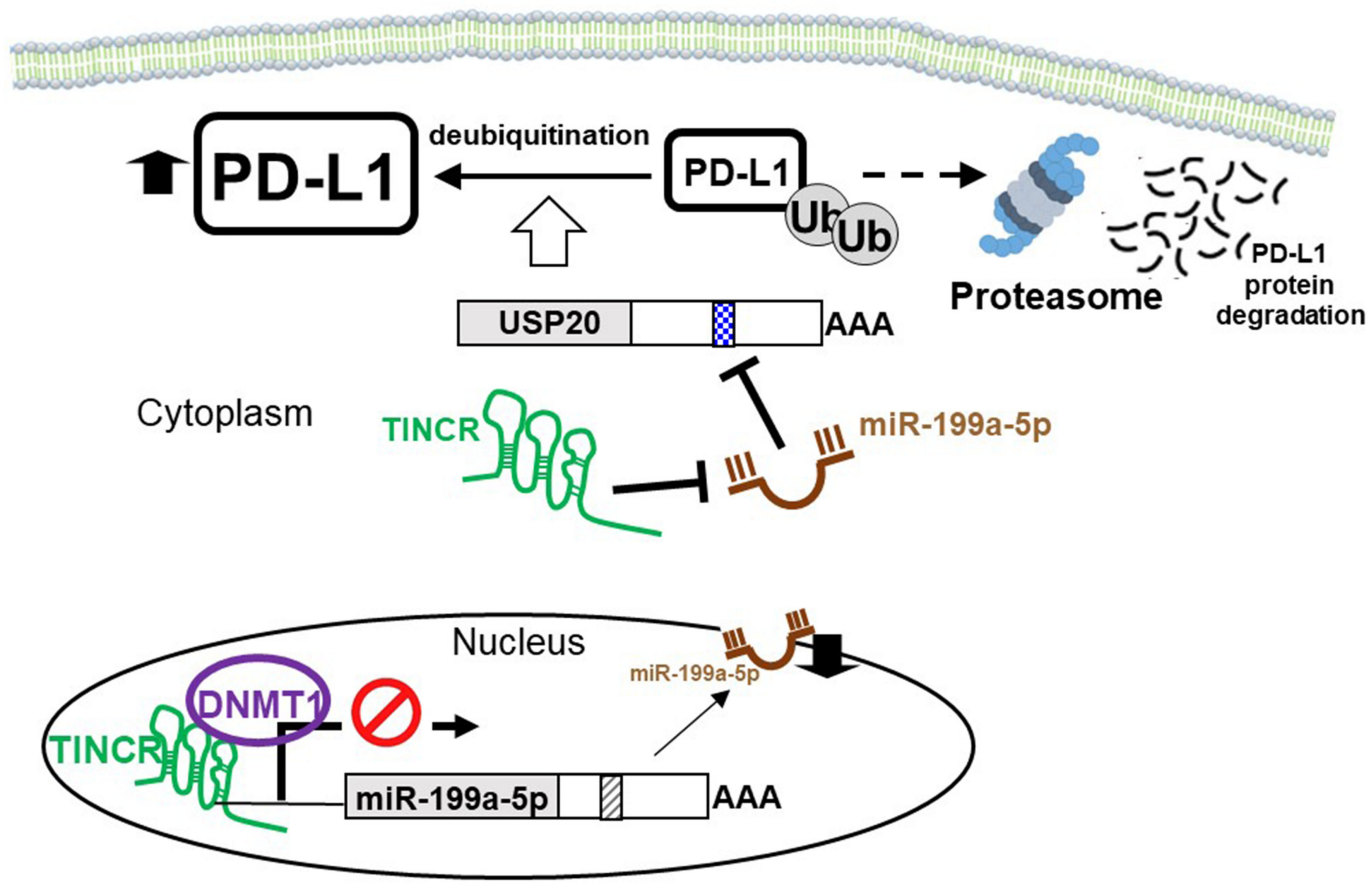
Schematic diagram showing a dual mechanism of PD-L1 regulation by a lncRNA TINCR in breast cancer to mediate resistance to cancer immunotherapy. TINCR was shown to increase PD-L1 expression by upregulating the USP20 via a dual mechanism. TINCR is expressed both in the nucleus and cytoplasm of breast cancer cells. In the cytoplasm, TINCR sponges miR-199a-5p and upregulates USP20 mRNA, thus increasing PD-L1 expression by suppressing its ubiquitination. In the nucleus, TINCR recruits DNMT1 to the gene promoter of miR-199-5p and promotes DNA methylation, thereby inhibiting the transcription of miR-199-5p. PD-L1: Programmed cell death ligand 1; lncRNA: long non-coding RNA; TINCR: tissue differentiation-inducing non-protein coding RNA; USP20: ubiquitin-specific protease 20.

PD-L1 expression can be regulated by lncRNAs that compete for miRNAs targeting PD-L1 mRNA, thereby promoting immune escape in various cancer types^[94]^. Table 4 summarizes representative ceRNA networks modulating PD-1/PD-L1 expression and their effects on cancer immunotherapy. Figure 3 illustrates the induction of cancer immunotherapy resistance due to ceRNA dysregulation-mediated PD-L1 upregulation in different cancer types. In breast cancer, the lncRNA tissue differentiation-inducing non-protein coding RNA (TINCR) was known to upregulate PD-L1 expression both *in vitro* and *in vivo*, thus promoting breast cancer progression. Importantly, TINCR knockdown was shown to significantly potentiate the antitumor efficacy of PD-L1 inhibitors in breast cancer in animal studies. Mechanistically, TINCR was shown to serve a dual function: it sequesters miR-199a-5p and inhibits its transcription, leading to increased PD-L1 expression [Figure 4]^[95]^. TINCR expression was detected in both the nucleus and cytoplasm of breast cancer cells. In the cytoplasm, TINCR was found to work as a molecular sponge of miR-199a-5p and upregulate the stability of the ubiquitin-specific protease 20 (USP20) mRNA via a ceRNA regulatory machinery, thus increasing PD-L1 expression by suppressing its ubiquitination^[95]^. In the nucleus, TINCR was found to recruit DNMT1 to the gene promoter of miR-199a-5p and promote DNA methylation, thereby inhibiting the transcription of miR-199-5p. Therefore, TINCR knockdown may be used as a novel strategy to potentiate PD-L1 immunotherapy in breast cancer. Similarly, CRC tumor cells have been reported to release exosomes enriched in the lncRNA KCNQ1OT1, which regulates the ubiquitination of PD-L1 via a miR-30a-5p/USP22 pathway to facilitate immune evasion^[96]^. According to TCGA database, the expressions of KCNQ1OT1 and miR-30a-5p are negatively correlated in tumor tissues. A high level of KCNQ1OT1 in CRC tumors was shown to sequester miR-30a-5p, thereby relieving the repression of the ubiquitin-specific peptidase USP22. It follows that PD-L1 expression is upregulated due to less ubiquitination, subsequently inhibiting CD8^+^ T cell response to induce CRC immune tolerance.

**Table 4:** Representative ceRNA networks modulating PD-1/PD-L1 expression and their effect on cancer immunotherapy

**Cancer type**	**Dysregulated ncRNA**	**miRNA involved**	**mRNA target of miRNA**	**Ref.**
BC	TINCR (lncRNA)	miR-199a-5p	USP20	[95]
CRC	MIR17HG (lncRNA)	miR-375	NF-κB/RELA	[156]
DLBCL	SNHG14 (lncRNA)	miR-5590-3p	ZEB1	[157]
HCC	RNAFOXD1-AS1 (lncRNA)	miR-615-3p	PI3K/AKT	[158]
NSCLC	hsa_circ_0020714 (circRNA)	miR-30a-5p	SOX4	[159]
OC	EMX2OS (lncRNA)	miR-654	AKT3	[160]
PC	PMSB8-AS1 (lncRNA)	miR-382-3p	STAT1	[161]
PCa	lncAMPC (lncRNA)	miR-637	JAK1-STAT3	[162]
Thy	RP11-424C20.2 (pseudogene)	miR-378a-3p	UHRF1	[163]
TNBC	GATA3-AS1 (lncRNA)	miR-676-3p	COPS5	[164]

ceRNA: Competing endogenous RNA; ncRNA: non-coding RNA; miRNA: microRNA; BC: breast cancer; CRC: colorectal cancer; DLBCL: diffuse large B cell lymphoma; HCC: hepatocellular carcinoma; NSCLC: non-small cell lung cancer; OC: ovarian cancer; PC: pancreatic cancer; PCa: prostate cancer; Thy: thymomas; TNBC: triple negative breast cancer.

In hepatocellular carcinoma (HCC), the lncRNA Lnc-CCNH-8 is highly expressed and correlates with poor prognosis^[97]^. Zhao *et al*. recently reported that Lnc-CCNH-8 could inactivate T cells *in vitro* and suppress antitumor immunity in immunocompetent mice *in vivo*^[97]^. Mechanistically, Lnc-CCNH-8 was shown to sequester miR-217 and, therefore, upregulate PD-L1 expression. Moreover, Lnc-CCNH-8 also stabilized PD-L1 via a miR-3173/Phakophilin 3 (PKP3) regulatory machinery. PKP3 is involved in deubiquitination of PD-L1. Therefore, overexpression of Lnc-CCNH-8 and PKP3 could upregulate PD-L1 levels in tumors. In addition, experimental mice bearing tumors with high Lnc-CCNH-8 expression are highly responsive to PD-L1 blockade treatment. Interestingly, HCC patients with high levels of plasma exosomal Lnc-CCNH-8 showed remarkably higher treatment responses to immune checkpoint inhibitors. Collectively, Lnc-CCNH-8 forms a novel ceRNA network to induce immune escape from CD8^+^ T cell-mediated cancer-killing effect by upregulating PD-L1 in a miR-217/miR-3173-dependent manner^[97]^. Critical players within the ceRNA network may be exploited as novel therapeutic targets to enhance PD-L1-based cancer immunotherapy. The plasma level of exosomal Lnc-CCNH-8 also represents a novel predictive marker for immunotherapy response in HCC.

In lung cancer, a ceRNA network has been constructed by using immune-related genes of LUAD samples from the TCGA database^[98]^. A 14-lncRNA immune-related signature was developed. In particular, the lncRNA C5orf64 was found to be positively correlated with the expression of immune checkpoint molecules (including PD-1, PD-L1, and CTLA-4) and immune cells (such as M2 macrophages, eosinophils, and neutrophils)) but negatively associated with the immunosuppressive Tregs and plasma cells. Thus, the lncRNA C5orf64 may be used as an indicator to predict the status of tumor microenvironment (TME) modulation^[98]^. The expression level of C5orf64 was found to be remarkably lower in LUAD tumor specimens than in non-tumorous tissues. Detailed ceRNA network analysis identified two potential pathways, C5orf64/miRNA-150/EREG and C5orf64/miRNA-155-ITK, which presumably play a critical role in shaping an immunosuppressive TME^[98]^. Further investigation is still needed to confirm the mechanistic explanation. The lncRNA FGD5-AS1 was reported to regulate PD-L1 expression by working as a miR-142 sponge, thereby promoting cancer cell proliferation, cisplatin resistance, migration, and tumor invasion^[99]^. On the other hand, the antisense lncRNA NKX2-1-AS1 was shown to downregulate PD-L1 by modulating NKX2-1 protein expression and reducing the cellular expression of cell adhesion molecules (including E-cadherin), thereby inhibiting cell migration in lung adenocarcinoma^[100]^. In NSCLC, the lncRNA MALAT1 was reported to upregulate PD-L1 by sequestering miR-200a-3p and promoting cancer propagation^[101]^. Therefore, FGD5-AS1/MALAT1 silencing or NKX2-1-AS1 ectopic expression may represent useful approaches to enhance PD-1/PD-L1 immunotherapy in lung cancer patients.

Furthermore, the dysregulation of circRNAs has also been reported to promote cancer development, migration, invasion, and immune evasion of NSCLC cells^[102]^. For example, the high expression of circFGFR1 (derived from FGFR1) in NSCLC tissues is associated with poor prognosis and clinical outcomes^[103]^. Mechanistically, circFGFR1 was shown to sequester miR-381-3p and increase the expression of its target gene CXCR4^[103]^. CXCR4 genetic silencing could sensitize NSCLC cells to PD-1 blockade immunotherapy^[103]^. To this end, CXCR4 is known to bind to CXCL12, subsequently increasing intracellular calcium content, cancer cell adhesion, proliferation, and gene transcription^[104,105]^. Hong *et al*. recently reported that circCPA4 and PD-L1 were overexpressed, but the miRNA let-7 was expressed at a lower level in NSCLC cell lines (than in normal bronchial epithelial cells) and tumor specimens (than in adjacent normal lung tissues)^[106]^. Importantly, NSCLC patients whose tumors expressed lower circCPA4 and PD-L1 levels but higher let-7 levels demonstrated a better prognosis. Detailed mechanistic investigation revealed that circCPA4 sponges let-7, thus upregulating PD-L1 to promote proliferation and EMT in NSCLC cells^[106]^. Moreover, circCPA4 was also shown to increase exosomal PD-L1 levels. In the co-culture system of NSCLC (H1299 or A549) and CD8^+^ T cells (isolated from human PBMCs), the depletion of circCPA4 was shown to reactivate CD8^+^ T cells. Interestingly, PD-L1 depletion was also shown to increase the levels of IFN-γ and IL-4, but reduce IL-10 expression in the CD8^+^ T cells and the supernatants, suggesting that the NSCLC cells inactivated CD8^+^ T cells in the co-culture via secreting PD-L1^[106]^. Collectively, circCPA4 increased PD-L1 expression in NSCLC cells by sponging let-7 to promote cancer stemness and cisplatin resistance, and also inactivated CD8^+^ T cells in the TME to facilitate immune evasion^[106]^.

Chen *et al*. recently reported the critical role played by the circRNA circNCOA3 in CRC immune escape and resistance to PD-1 blockade therapy^[107]^. In CRC patient tumors, circNCOA3 was found to be remarkably overexpressed in samples resistant to anti-PD-1 monoclonal antibodies. High circNCOA3 was significantly correlated with worse survival and poor treatment outcomes in CRC patients. In cell line studies, the knockdown of circNCOA3 was shown to suppress cancer cell proliferation and invasion. In animal studies, circNCOA3 knockdown was found to increase the proportion of anticancer CD8^+^ T cells but reduce the abundance of the immunosuppressive myeloid-derived suppressor cells (MDSCs). Importantly, circNCOA3 knockdown was reported to retard tumor growth and potentiate the antitumor efficacy of PD-1 blockade immunotherapy in tumor-bearing mouse models^[107]^. Subsequent mechanistic investigation revealed that circNCOA3 acted as a ceRNA for miR-203-3p to modulate CXCL1 expression. To this end, CXCL2 derived from the M2 macrophages, upon binding to CXCR2, has been shown to activate the PI3K/AKT/NF-B signaling pathway to increase PD-L1 expression. circNCOA3 may be further developed into a useful biomarker to predict the response and prognosis of CRC patients upon PD-1 blockade therapy.

In pancreatic cancer, hypoxia is known to induce HIF1, ADAM10, and sMICA, thus leading to reduced NKG2D in natural killer (NK) cells and immune escape of tumor cells^[108]^. The circRNA circ_0000977 was induced by hypoxia. Knockdown of circ_0000977 was shown to potentiate NK cell-mediated lysis of cancer cells under hypoxic conditions in a HIF1- and ADAM10-dependent manner. Both HIF1α and ADAM10 are direct downstream targets of miR-153. circ_0000977 was shown to sequester miR-153 and relieve the repression of HIF1α and ADAM10 mRNA in pancreatic cancer cell line Panc-1. Collectively, the circ_0000977/miR-153/HIF1α/ADAM10 ceRNA network represents a novel mechanism contributing to the hypoxia-mediated immune escape of pancreatic cancer cells^[108]^.

While there is growing interest in understanding the role of ceRNA regulation in cancer immunotherapy, research in this domain is still in its infancy. ceRNA regulation may also affect the expression of other immune-related genes regulating the TME, tumor infiltration of immune cells, and biological functions of the immune cells. By modulating the expression of genes involved in various immune response pathways, ceRNAs could indirectly regulate the efficacy of cancer immunotherapy. It is noteworthy that the specific ceRNA networks and their impact on the various immune checkpoints may vary across different cancer types and in different contexts. Further research is needed to fully understand the complexity of ceRNA-mediated regulation of the immune checkpoints and its implications on cancer immunotherapy.

## CONCLUSION AND FUTURE PERSPECTIVES

Understanding the ceRNA networks and their role in anticancer drug resistance could yield novel insights for the development of effective therapeutic strategies for treating refractory tumors and useful prognostic biomarkers for predicting clinical outcomes. By targeting specific ceRNAs or manipulating the ceRNA network, it may be possible to overcome drug resistance and improve the effectiveness of cancer treatments. Interestingly, flavonoids (bioactive polyphenolic compounds abundant in fruits, vegetables, and many medicinal plants) have been reported to regulate cancer-related genes via the ceRNA network, thereby inhibiting cancer growth and reversing chemoresistance^[109]^. In gastric cancer, chrysin has been shown to promote apoptosis via the H19/miR-let-7a/COPB2 regulatory pathway^[110]^. Another extensively studied flavonoid, quercetin, was reported to inhibit cancer proliferation and tumor invasion by upregulating miR-146a in breast cancer^[111]^ and sensitize NSCLC cells to radiotherapy by modulating the miR-16-5p/WEE1 axis^[112]^. These ceRNA-modulating flavonoids may be combined with anticancer drugs to potentiate the therapeutic effect and overcome cancer drug resistance. Moreover, the ceRNA components involved may be exploited as useful biomarkers to select the patient population who could respond better to the drug combination.

Recent advances in high-throughput sequencing techniques have enabled the discovery of numerous biomarkers, including protein-coding RNAs and ncRNAs, from tissue and blood samples of cancer patients. To this end, most existing cancer biomarkers are solely relying on gene expression patterns. However, the expression patterns are not able to reflect the underlying interactions/mechanisms^[113,114]^. Therefore, gene expression signatures for patient populations harboring the same clinical condition reported by different research teams are usually highly heterogeneous^[114]^. To this end, system-guided ceRNA network analysis may represent a more reliable method for biomarker identification and the generated ceRNA signature may also facilitate precision medicine. The systematic method of computational approaches for ceRNA network-driven biomarker discovery has been recently reviewed^[115,116]^. Clinical drug response analyses are usually performed to validate ceRNA biomarkers associated with anticancer drug responses^[117]^.

Components of ceRNA networks, including miRNAs, lncRNAs, and circRNAs, have been considered emerging biomarkers for cancer diagnosis, prognosis prediction, and treatment monitoring^[118-120]^. Using a ceRNA network-driven method, a ceRNA network comprising 12 lncRNAs, 2 miRNAs, and 15 mRNAs was identified as a prognostic biomarker to predict survival for patients with pancreatic adenocarcinoma^[121]^. For breast cancer, Wang *et al*. recently reported a lncRNA H19- and BRCA1/2-associated ceRNA signature that could distinguish patients with favorable versus dismal survival outcomes^[122]^. For gastric cancer, Sui *et al*. identified a 2-lncRNA signature within a ceRNA network as a prognostic biomarker for predicting patient survival^[123]^. The therapeutic significance is that genetic silencing of the two lncRNAs (LINC01644 and LINC01697) was shown to effectively inhibit gastric cancer cell proliferation. For lung cancer, a lung squamous cell carcinoma-specific ceRNA network has been recently constructed using TCGA RNA-sequencing datasets^[123]^. A 2-lncRNA signature (consisting of FM06P and PRR26) was identified as a prognostic biomarker for overall patient survival^[123]^. Moreover, ceRNA biomarkers have also been reported to predict and monitor anticancer drug response in the clinic. By integrating the expression profiles of lncRNA, miRNA, and mRNA from a pan-cancer ceRNA network with the patient survival data after anticancer drug treatment, Qi *et al*. identified a signature of drug response-related ceRNA (DRCE) that was significantly correlated with individual drug response to cisplatin^[116]^. Moreover, they also identified two DRCEs (NEAT1/hsa-miR-130b/TP53INP1 and NEAT1/hsa-miR-18a/NBR1) capable of modulating the anticancer efficacy of tamoxifen in breast cancer patients harboring TP53 mutation^[116]^. Most recently, for lung cancer, Liao *et al*. reported a novel prognostic ceRNA network biomarker consisting of RGN (inhibitory protein of calcium signaling) and its related miRNA (hsa-miR-203a-3p) and two lncRNAs (ZNF876P and PSMG3-AS1)^[124]^. The high RGN expression group was found to be associated with lower cancer immunotherapy efficacy and prognosis, which was consistent with an immunosuppressive tumor microenvironment^[125]^.

With the increasing number of experimentally validated ceRNA networks reported in recent years, a few databases have been developed to compile experimentally supported ceRNA interactions with comprehensive annotations [Table 5]. A few recently developed ceRNA databases also include patient demographics and clinical drug response data, thus making them more clinically relevant and potentially allowing for personalized prediction of ceRNA modulation outcomes. LncACTdb 3.0 is a comprehensive database of experimentally validated ceRNA interactions across 25 species and 537 diseases^[126]^. It also compiled the lncRNA/mRNA/miRNA expression profiles with clinical and pathological information extracted from 62 datasets in TCGA and GEO. Computational tools are available for exploring the effects of ceRNA on individuals with specific pathological backgrounds.

**Table 5:** Representative databases/online resources for ceRNAs relevant to anticancer drug resistance

**Database**	**Online accession**	**Characteristic features**	**Ref.**
Cupid	https://cupidtool.sourceforge.net/ (accessed on 22 May 2024)	● Online tool for simultaneous prediction of miRNA target interactions and their mediated ceRNA interactions	[6]
DIANA-LncBase v3.0	www.microrna.gr/LncBase (accessed on 22 May 2024)	● Collecting the correlations of miRNA-lncRNA pairs, and profiles of lncRNA expression in different cell types and organ tissues	[165]
ExoceRNA atlas	https://ngdc.cncb.ac.cn/databasecommons/database/id/7334 (accessed on 22 May 2024)	● Composing cancer ceRNAs in human blood exosomes	[166]
lnCeDB	http://gyanxet-beta.com/lncedb (accessed on 22 May 2024)	● Listing human lncRNAs that may act as ceRNAs	[167]
Linc2GO	https://pubmed.ncbi.nlm.nih.gov/23793747/ (accessed on 22 May 2024)	● A human lincRNA function annotation resource based on ceRNA hypothesis ● Composing miRNA-lincRNA and miRNA-mRNA interaction data	[168]
LncACTdb 3.0	http://www.bio-bigdata.hrbmu.edu.cn/LncACTdb (accessed on 22 May 2024)	● Listing comprehensive information of ceRNAs in different species and under different disease states	[125]
LnCeVar	https://ngdc.cncb.ac.cn/databasecommons/database/id/6187 (accessed on 22 May 2024)	● Compiling genomic variations that may disrupt ceRNA network ● Curated from high-throughput sequencing datasets or published literature	[127]
LnCeCell	http://www.bio-bigdata.hrbmu.edu.cn/LnCeCell/ (accessed on 22 May 2024)	● Collecting cell-specific lncRNA-associated ceRNA networks, applicable for personalized characterization of diseases	[126]
miRSponge	https://bio.tools/mirsponge (accessed on 22 May 2024)	● Composing experimentally supported miRNA sponges and ceRNA networks	[169]
SomamiR 2.0	http://compbio.uthsc.edu/SomamiR (accessed on 22 May 2024)	● Compiling cancer somatic mutations in miRNA that may disrupt the interactions between miRNAs and ceRNA (circRNA, lncRNA, and mRNA)	[170]
StarBase v2.0	http://starbase.sysu.edu.cn/ (accessed on 22 May 2024)	● Collecting experimentally supported miRNA-lncRNA and miRNA-mRNA interactions ● Curated from CLIP-Seq data available in published literature	[171]

ceRNA: Competing endogenous RNA; miRNA: microRNA; lncRNA: long non-coding RNA; lincRNA: long intergenic non-coding RNA.

LnCeCell is another database of predicted lncRNA-associated ceRNA networks constructed at the single-cell resolution^[127]^. It is handpicked from cellular-specific ceRNA regulations and functional status of more than 94,000 cells in 25 tumor types. The database compiles more than 9,000 experimentally validated lncRNA biomarkers, associated with drug resistance, prognosis, tumor metastasis, and recurrence. The unique feature of LnCeCell is that it provides a global map of ceRNA sub-cellular localization at a single cancer cell level, which was manually curated from the original data sources.

LnCeVar is another recently developed database of genomic variations that disturb ceRNA network regulation^[128]^. It curated genomic variations-ceRNA events from patient samples and cell lines. Of relevance to anticancer drug response, the database contains more than 2,000 experimentally validated circulating, drug-resistant and prognosis-related lnRNA biomarkers. A few user-friendly searching and browsing interfaces are available for retrieval and analysis of data^[128]^. In particular, LnCeVar-Survival can be used to conduct COX regression analyses and produce patient survival curves for specific genomic variation-ceRNA events. Thus, LnCeVar represents a useful tool for investigating the influence of personalized genomic variations that disturb ceRNA network in various diseases, including drug-refractory cancers.

It is noteworthy that the specific impact of disrupting ceRNA networks on cancer drug response may vary depending on the specific ceRNAs involved, the miRNAs and target genes regulated by the ceRNAs, and the cellular context. Subcellular localization and abundance of the ceRNA(s), and their interaction with other cellular pathways will also affect the biological outcomes of the ceRNA network. As the non-coding regions encoding regulatory RNAs make up close to 99% of the human genome, genomic alterations in cancer could have substantial effects on ceRNA networks that are largely regulated by ncRNAs. To this end, the intersection between cancer genomic alterations and ceRNA regulation has been unappreciated.

Moreover, an interplay between ceRNA network and epigenetic regulation of the miRNA components has been proposed^[129]^. This is exemplified by the ceRNA network operating in the estrogen receptor signaling pathway. In ovarian cancer, miR-193a is known to target E2F6 (a downstream target of estrogen receptor), c-KIT (a widely-studied marker for cancer stemness), and PBX1 (a transcriptional activator for the immunosuppressive cytokine IL-10). Interestingly, epigenetic silencing of miR-193a by the E2F6 protein was shown to be required to upregulate c-KIT and PBX1 mRNA, in order to promote cancer stemness and immune evasion^[130]^. Further research is warranted to fully unravel the complexity of ceRNA-mediated regulation and its implications on drug resistance.

## DECLARATIONS

### Copyright

© The Author(s) 2024.
